# Graft Uptake Following Myringoplasty With Adjuvant Autologous Platelet-Rich Plasma: A Prospective, Non-randomized Comparative Cohort Study in Tiruvannamalai District, Tamil Nadu, South India

**DOI:** 10.7759/cureus.117279

**Published:** 2026-10-01

**Authors:** Neethu Sara George, Arun Kumar G, Liju Rajan, Neeraj V Mohandas

**Affiliations:** 1 Otorhinolaryngology, Arunai Medical College and Hospital, Tiruvannamalai, IND; 2 Community Medicine, Dhanalakshmi Srinivasan Medical College and Hospital, Perambalur, IND

**Keywords:** chronic suppurative otitis media, graft uptake, hearing outcome, myringoplasty, platelet-rich plasma, pure-tone audiometry, tympanic membrane perforation

## Abstract

Background

Tympanic membrane (TM) perforation may require surgical repair to restore anatomical integrity and hearing. Autologous platelet-rich plasma (PRP) has been used as an adjunct to myringoplasty to support tissue healing and graft uptake. This study was conducted to evaluate graft uptake following myringoplasty with adjunctive autologous PRP and to compare postoperative hearing outcomes between PRP and non-PRP cohorts.

Materials and methods

A prospective, non-randomized comparative cohort study was conducted among 174 patients undergoing myringoplasty at a tertiary care teaching hospital and medical college in Tiruvannamalai district, Tamil Nadu, South India. Participants were classified into PRP and non-PRP cohorts according to participant willingness and operating surgeon preference, with 87 patients in each cohort. Successful graft uptake was defined as an intact TM without persistent, residual, or recurrent perforation at three months. Hearing outcome was assessed using pure-tone audiometry (PTA), and PTA gain was calculated from preoperative and postoperative values. Associations with graft uptake were examined using modified Poisson regression with robust variance. Postoperative PTA was additionally assessed using analysis of covariance (ANCOVA) to account for baseline differences. A p-value <0.05 was considered statistically significant.

Results

The PRP cohort had a mean (± standard deviation (SD)) age of 34.64 (± 7.22) years, compared with 35.11 (± 8.51) years in the non-PRP cohort. At three months, graft uptake was achieved in 76 (87.4%) patients in the PRP cohort and 68 (78.2%) patients in the non-PRP cohort. After adjustment, PRP remained associated with higher graft uptake, but the association was not statistically significant (adjusted risk ratio (RR): 1.13; 95% confidence interval (CI): 0.98-1.26). Operated-ear PTA improved significantly after surgery, with a mean improvement of 8.81 dB (95% CI: 7.93-9.69; p < 0.001). PTA gain did not differ significantly between the PRP and non-PRP cohorts (9.47 vs 8.15 dB; p = 0.138). After adjustment for baseline PTA, postoperative hearing also showed no significant between-cohort difference (p = 0.662). Patients with successful graft uptake demonstrated greater PTA improvement than those with graft failure (10.42 vs 1.07 dB; p < 0.001).

Conclusion

Adjunctive autologous PRP was associated with a numerically higher three-month graft uptake rate, although the adjusted association was not statistically significant. Hearing improved significantly following myringoplasty in both cohorts. No statistically significant difference in postoperative hearing was observed between the PRP and non-PRP cohorts. Larger randomized clinical trials are needed to clarify the clinical value of PRP in myringoplasty.

## Introduction

Tympanic membrane (TM) perforation is a common consequence of chronic middle-ear disease, trauma, and other otologic conditions [1]. Persistent perforation may result in recurrent otorrhea, conductive hearing loss, and impaired protection of the middle ear from the external environment [2].

Myringoplasty remains an established surgical treatment for persistent TM perforation. Its principal objectives are anatomical closure of the perforation and improvement of hearing [3]. However, despite the advances in surgical techniques, graft failure after myringoplasty remains clinically relevant. Reported outcomes vary according to patient characteristics, perforation size, middle-ear status, graft material, and surgical factors. This variability has encouraged interest in biological approaches to improve graft healing [4].

Platelet-rich plasma (PRP) is an autologous platelet concentrate containing multiple growth factors involved in tissue repair, angiogenesis, cellular proliferation, and wound healing [5]. Applied during myringoplasty, PRP may provide a local biological environment that supports graft integration and TM regeneration. Its autologous nature also makes it an attractive adjunct because it avoids exposure to allogeneic biological products [6].

Evidence regarding the clinical benefit of PRP in myringoplasty has progressively expanded, although the findings have not been entirely consistent [7]. Several comparative studies have reported higher graft uptake and improved postoperative healing with platelet concentrates, suggesting a potential benefit when PRP is used as an adjunct to conventional myringoplasty [6]. However, other controlled studies have failed to demonstrate a significant advantage of PRP in anatomical closure or hearing outcomes. In a randomized trial conducted by Sharma et al. in 2021, PRP-assisted surgery produced graft uptake comparable to conventional myringoplasty, with no significant difference in postoperative hearing improvement [8].

The apparent benefit of PRP in myringoplasty therefore remains clinically relevant, but its magnitude is not yet firmly established. Differences in perforation characteristics, graft materials, surgical techniques, PRP preparation methods, application protocols, and duration of follow-up may contribute to the variation between studies [9].

Hence, this prospective, non-randomized comparative cohort study was conducted in Tiruvannamalai district of Tamil Nadu, South India, to evaluate whether adjunctive autologous PRP is associated with improved graft uptake following myringoplasty. Postoperative hearing outcomes were also assessed to determine whether any anatomical benefit was accompanied by measurable functional improvement.

## Materials and methods

Study design and setting

A prospective, non-randomized comparative cohort study was conducted among patients undergoing myringoplasty for TM perforation. The study was undertaken in the Department of Otorhinolaryngology at a tertiary care hospital and medical college located in Tiruvannamalai district, Tamil Nadu, South India. The study was conducted over a period of 12 months from August 2025 to July 2026, with the last study participant being recruited in April 2026.

Study population and eligibility criteria

The study participants were recruited using a non-probability consecutive sampling approach during the study period. The study participants comprised patients clinically diagnosed with tubo-tympanic chronic suppurative otitis media (CSOM) for whom myringoplasty was planned. The diagnosis of CSOM was made according to departmental protocol, and it was primarily based on characteristic history and otoscopic findings [10]. Patients were screened clinically before surgery and assessed against the predefined eligibility criteria.

Participants aged 18-60 years who were willing to undergo surgery and complete postoperative follow-up were considered eligible. Only patients with a dry ear for at least six weeks were included. TM perforations other than attic and marginal perforations were considered suitable for inclusion. Patients with a mixed hearing loss were eligible if they otherwise met the inclusion criteria. Patients were also required to have clinically normal eustachian tube function.

Patients were excluded if they had any of the following: (1) an actively discharging ear, (2) cholesteatoma, (3) isolated sensorineural hearing loss (SNHL), (4) any immunocompromised condition including uncontrolled diabetes mellitus, (5) any ossicular erosion, (6) retracted TM, and (7) previous history of ear surgery done in the same ear.

Sample size estimation

The sample size was estimated using a two-independent-proportions framework for comparison of graft uptake between the PRP and non-PRP cohorts. As no single directly comparable study provided both expected proportions under the same study conditions, literature-derived estimates were used from previously published myringoplasty studies.

A graft uptake rate of 94.4% was considered for the PRP cohort, as reported by Sharma et al. [8], while an expected graft uptake rate of 80% for conventional myringoplasty was derived from Kamath et al. [3].

A two-sided significance level of 5% and statistical power of 80% were assumed, with equal allocation between the two cohorts. Using the two-independent-proportions approach based on Cohen's h in the software G*Power (version 3.1; The G*Power Team, Germany) [11], the minimum required sample size per cohort, allowing for an anticipated loss to follow-up of 10%, was calculated as 87. The study, therefore, included 87 participants in each cohort, giving a total sample size of 174.

Study procedure

A total of 232 study participants were screened for eligibility, of whom 58 were excluded. Eligible study participants were enrolled before surgery and followed prospectively after myringoplasty. The enrolled study participants were categorized into two cohorts according to the use of autologous PRP during surgery. Both methods of myringoplasty were routinely done by the surgeons in the Department of Otorhinolaryngology at this hospital.

Allocation of PRP

Treatment allocation was non-randomized. The decision to use adjunctive PRP was based on participant willingness and operating surgeon preference. No formal randomization, investigator-assigned allocation, or additional standardized allocation criteria were used.

The study participants were subsequently classified into the PRP and Non-PRP cohorts. Hence, out of the total of 174 study participants, 87 underwent myringoplasty with intraoperative application of autologous PRP, while 87 participants underwent conventional myringoplasty without PRP. The flow diagram of the study is depicted in Figure 1.

**Figure 1:**
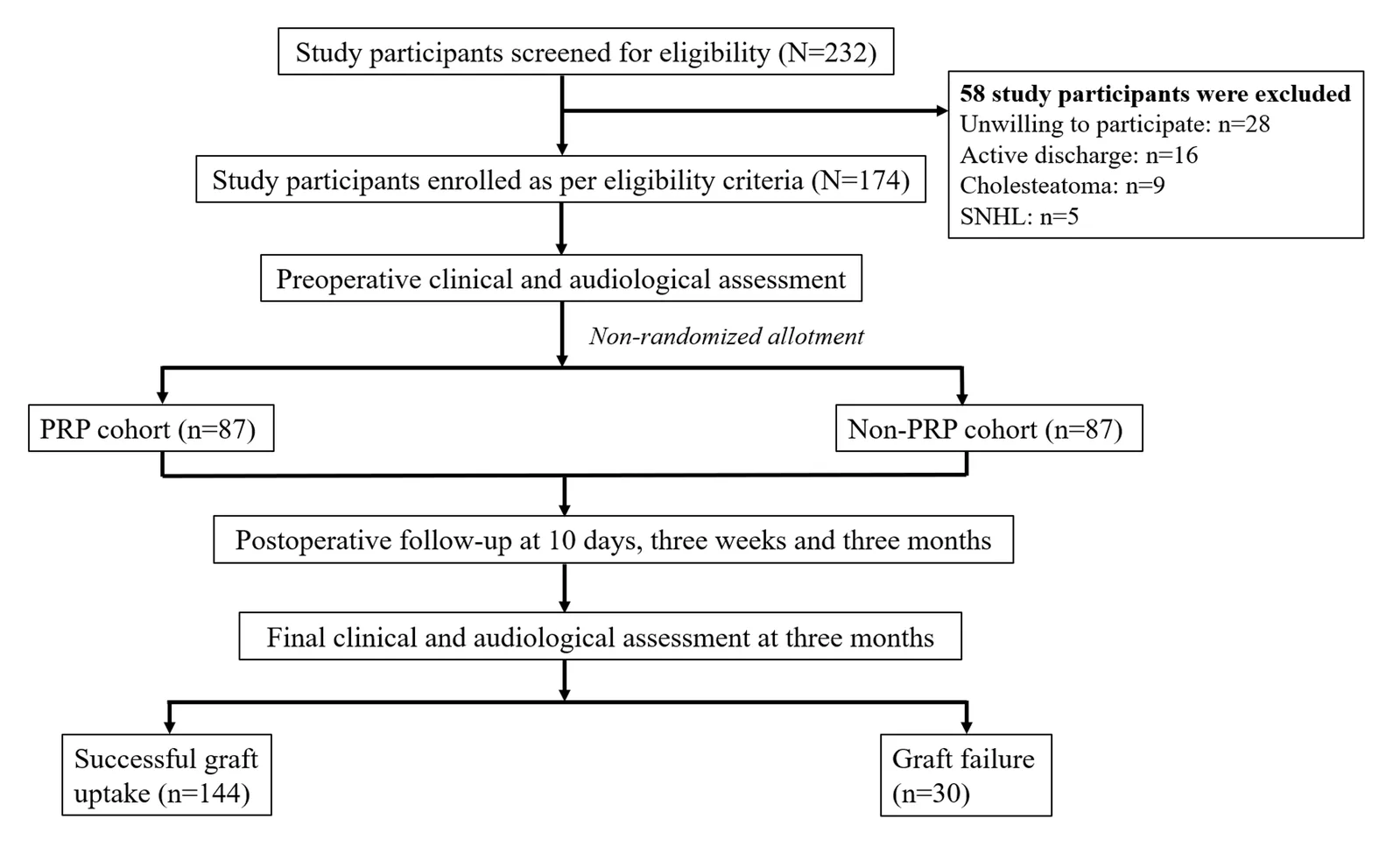
Flow diagram of the study SNHL: Sensorineural hearing loss; PRP: Platelet-rich plasma Image created by the authors using Microsoft PowerPoint 2021 (Microsoft® Corp., Redmond, WA)

Preoperative clinical assessment

All study participants underwent a detailed otorhinolaryngological assessment before surgery. Demographic characteristics and relevant clinical findings were documented using a structured data collection format.

The recorded variables included age, gender, ear-related symptoms, perforation characteristics, and preoperative and postoperative audiological findings. The presence of ear pain, tinnitus, and hearing difficulty was also documented after medical examination.

The affected ear was recorded as right or left. TM perforations were measured using an otoscope and categorized according to the documented perforation size as small, medium, or large. Small perforations involved one quadrant, medium perforations involved two quadrants, and large perforations involved three or four quadrants of the pars tensa of the TM [12]. Preoperative assessment also included evaluation of the middle ear and Eustachian tube function according to routine departmental protocol.

Audiological assessment

Preoperative hearing assessment was performed using bedside tuning-fork tests followed by pure-tone audiometry (PTA) as part of the routine assessment before myringoplasty. The preoperative audiological findings were used to characterize hearing status before surgery. Postoperative PTA was also done at three months to assess hearing improvement.

A 512-Hz tuning fork was used for the Rinne and Weber tests. Both tuning-fork test findings were interpreted together rather than independently. As tuning-fork tests have limited sensitivity for smaller conductive components and may not reliably distinguish mixed from conductive hearing loss, the definitive classification of hearing loss was based on PTA.

PTA served as the primary tool for identifying the degree and type of hearing loss. Air- and bone-conduction thresholds were assessed according to standard audiometric procedures. Air-conduction thresholds were measured at 500, 1,000, 2,000, 4,000, 6,000, and 8,000 Hz, while bone-conduction thresholds were assessed at the relevant frequencies [13]. Hearing loss was classified as conductive, sensorineural, or mixed based on the presence or absence of an air-bone gap in conjunction with the audiometric configuration [14]. PTA was conducted using an Equinox model designed by Interacoustics.

Hearing impairment was defined based on the four-frequency pure-tone average, calculated as the mean hearing threshold at 500 Hz, 1,000 Hz, 2,000 Hz, and 4,000 Hz [15]. A pure-tone average of ≥25 dB was considered hearing impairment, which included conductive, sensorineural, as well as mixed hearing loss [16]. However, none of the patients in this study had sensorineural hearing loss.

Myringoplasty procedure

All study participants underwent myringoplasty using the underlay technique with temporalis fascia, according to the standard surgical protocol followed by multiple surgeons in the Department of Otorhinolaryngology. TM perforation was evaluated before graft placement. Standard perioperative precautions and postoperative care were provided to all the study participants of both cohorts. For the study participants with bilateral TM perforation, the procedure was done for the clinically worse side first, and only the ear undergoing surgery during the study period was considered for operative and audiological outcome assessment in this study. The principal difference between the cohorts was the application of autologous PRP in the PRP cohort.

Preparation and application of autologous platelet-rich plasma for the PRP cohort

Autologous PRP was prepared in the National Accreditation Board for Testing and Calibration Laboratories (NABL)-accredited hospital laboratory, which is supervised by the Department of Biochemistry, following standard protocols.

The PRP was prepared using peripheral venous blood (20-60 mL of whole blood depending on the amount of PRP required for the procedure) collected from the participant under sterile conditions. Blood was collected into tubes containing acid citrate dextrose as an anticoagulant. The collected blood was initially subjected to a low-speed centrifugation step. The plasma fraction containing platelets was then transferred into a sterile tube. A second centrifugation step was performed at a higher speed to concentrate the platelets, following which the platelet-poor plasma fraction was removed. The concentrated platelet fraction was gently resuspended in a minimal volume of plasma [17].

The resulting preparation was used as autologous PRP and transferred to the operating theatre and applied over the graft surface during myringoplasty for the PRP cohort. Participants in the non-PRP cohort underwent the same surgical procedure without PRP application.

Postoperative follow-up

The study participants were followed prospectively after surgery. Three postoperative assessments were conducted at approximately 10 days, three weeks, and three months. All the study participants of both cohorts completed all three postoperative assessments with no loss to follow-up, as they were enrolled in a special medical scheme of the hospital which entitled them to postoperative assessments at no additional cost.

During all postoperative follow-ups, the operated tympanic membrane was examined for graft healing and anatomical closure. Postoperative graft status was assessed during the scheduled clinical follow-up without blinding. PTA was performed at three months as part of the scheduled postoperative assessment.

Primary and secondary clinical outcomes

The final scheduled postoperative assessment at three months was used for determination of the primary clinical outcome, which was the successful graft uptake following myringoplasty. Successful graft uptake was defined as an intact TM without persistent, residual, or recurrent perforation at the final postoperative assessment at the end of three months [18]. Graft failure was defined as persistence or recurrence of TM perforation at three months [18].

The secondary clinical outcome was change in operated-ear hearing threshold following surgery, which was assessed by PTA gain. The PTA gain was calculated as the difference between the preoperative and postoperative PTA values.

Statistical analysis

The collected data were entered into Microsoft Excel (Microsoft® Corp., Redmond, WA), appropriately coded, and analysed using Statistical Product and Service Solutions (SPSS, version 26.0; IBM SPSS Statistics for Windows, Armonk, NY). Continuous variables were summarized using the mean and standard deviation when normally distributed. Categorical variables were presented as frequencies and percentages. Distributional assumptions for continuous variables were assessed using graphical methods and tests of normality. Categorical variables were compared using the chi-square test. Independent-samples t-tests were used for normally distributed continuous variables. Preoperative and postoperative measurements were compared using a paired-samples t-test. As baseline operated-ear PTA differed between the treatment cohorts, postoperative PTA was additionally compared using analysis of covariance (ANCOVA), with preoperative PTA included as a covariate.

As allocation of the study participants was non-randomized, multivariable analysis was done to account for clinically relevant confounding. Covariates were selected a priori based on clinical relevance and established prognostic factors for graft uptake, with consideration of variables available in the study dataset. As the number of graft failures was limited, the final model was restricted to a parsimonious set of clinically relevant covariates. A modified Poisson regression model with robust variance estimation was used to estimate the adjusted risk ratio (RR) for successful graft uptake. Sensitivity analysis was performed using a multivariable logistic regression model and was reported as an adjusted odds ratio (OR).

The primary outcome was expressed as the proportion of participants achieving successful graft uptake in each cohort. The association between adjunctive PRP use and successful graft uptake was quantified using the RR with its corresponding 95% confidence interval. All statistical tests were two-sided. A p-value of < 0.05 was considered statistically significant. Effect estimates were reported with corresponding 95% confidence intervals.

Ethical considerations

Ethical approval was obtained from the Institutional Ethics Committee of Arunai Medical College and Hospital (IEC/022/3881/2025) before commencement of the study. Written informed consent was obtained from all participants before enrolment and surgery.

Autologous PRP was prepared from the participant's own peripheral blood. Appropriate sterile precautions were followed during blood collection, processing, and application. Participants were informed about the surgical procedure, PRP intervention, expected benefits, and potential risks before providing consent.

## Results

A total of 174 patients undergoing myringoplasty for TM perforation were included in the final analysis. The study participants in the PRP cohort (myringoplasty with PRP) had a mean (± SD) age of 34.64 (± 7.22) years, while the study participants in the non-PRP cohort (myringoplasty without PRP) had a mean (± SD) age of 35.11 (± 8.51) years, with ages ranging from 18 to 60 years in both cohorts. All participants reported hearing difficulty, while none had active ear discharge or giddiness at enrolment. The distribution of age, gender, ear pain, and tinnitus was comparable between the PRP and non-PRP cohorts, with no statistically significant differences observed for any of these baseline characteristics. The distributions of perforation side, perforation size, tuning-fork findings, and operated side were similar between the PRP and non-PRP cohorts (all p > 0.05). However, preoperative operated-ear PTA was higher in the PRP cohort than in the non-PRP cohort (45.28 ± 8.48 vs 42.22 ± 8.77 dB; p = 0.02). The basic characteristics and medical details of the study participants are summarized in Table 1.

**Table 1:** Baseline demographic and clinical characteristics of the study participants according to the treatment cohort (N = 174) *p-value < 0.05 was considered statistically significant. The chi-square test was used for categorical variables. Test statistics: age, ꭓ2 = 0.57; gender, χ² = 0.10; ear pain, χ² = 0.39; tinnitus, χ² = 0.05; side of tympanic membrane perforation, χ² = 1.28; perforation size, χ² = 1.52; tuning-fork findings, χ² = 0.10; site of procedure, χ² = 1.15. #Preoperative PTA was compared using an independent-samples t-test, t(172) = 2.34.

Sl. No.	Baseline characteristics	Myringoplasty with PRP (N=87)		Myringoplasty without PRP (N=87)		p-value
		Frequency (n)	Percentage (%)	Frequency (n)	Percentage (%)	
1.	Age (in years)					
	18-25	11	12.6	12	13.8	0.75
	26-40	56	64.4	59	67.8	
	>40	20	23	16	18.4	
2.	Gender					
	Male	33	37.9	35	40.2	0.75
	Female	54	62.1	52	59.8	
3.	Ear pain					
	Yes	56	64.4	52	59.8	0.53
	No	31	35.6	35	40.2	
4.	Tinnitus					
	Yes	12	13.8	11	12.6	0.82
	No	75	86.2	76	87.4	
5.	Side of tympanic membrane perforation					
	Right	29	33.3	35	40.2	0.52
	Left	44	50.6	42	48.3	
	Bilateral	14	16.1	10	11.5	
6.	Size of tympanic membrane perforation					
	Small	31	35.6	31	35.6	0.46
	Medium	36	41.4	42	48.3	
	Large	20	23	14	16.1	
7.	Tuning fork test results					
	Conductive hearing loss	56	64.4	58	66.7	0.75
	Mixed hearing loss	31	35.6	29	33.3	
8.	Site of procedure (even in bilateral tympanic membrane perforation)					
	Right ear	34	39.1	41	47.1	0.28
	Left ear	53	60.9	46	52.9	
Preoperative PTA (Mean ± SD)^ #^		45.28 ± 8.48		42.22 ± 8.77		0.02*
		Mean difference (95% CI): 3.05 (0.47-5.64)				

At three months, graft uptake was successful in 76 of 87 patients (87.4%) in the PRP cohort and 68 of 87 patients (78.2%) in the non-PRP cohort. The crude risk ratio for successful graft uptake with PRP compared with no PRP was 1.12 (95% CI: 0.97-1.28), with no statistically significant difference between the cohorts. The absolute difference between the two cohorts is 9.2 percentage points (Table 2).

**Table 2:** Comparison of graft uptake at three months according to the treatment cohort (N = 174) *p-value < 0.05 was considered statistically significant. The chi-square test was used; χ² = 2.58 PRP: Platelet-rich plasma

Outcome	Myringoplasty with PRP (N=87) n (%)	Myringoplasty without PRP (N=87) n (%)	Crude Risk Ratio (95 % CI)	p-value
Successful graft uptake	76 (87.36)	68 (78.16)	1.12 (0.97-1.28)	0.10
Graft failure	11 (12.64)	19 (21.84)		

In the multivariable modified Poisson regression analysis, treatment cohort, perforation size, age, and preoperative operated-ear PTA were evaluated as potential factors associated with successful graft uptake (Table 3). Compared with no PRP, adjunctive PRP was associated with a higher probability of successful graft uptake, although the adjusted association did not reach statistical significance. Neither perforation size, age, nor preoperative PTA showed a statistically significant independent association with successful graft uptake.

**Table 3:** Factors associated with successful graft uptake at three months (N = 174) *p-value < 0.05 was considered statistically significant. The modified Poisson regression model was used.

Sl. No.	Variables	Successful graft uptake		Unadjusted RR (95% CI)	Adjusted RR (95% CI)
		Yes n (%)	No n (%)		
1.	Treatment cohort				
	Myringoplasty without PRP	68 (78.2)	19 (21.8)	1	1
	Myringoplasty with PRP	76 (87.4)	11 (12.6)	1.12 (0.97-1.28)	1.13 (0.98-1.26)
2.	Perforation size				
	Small	51 (82.3)	11 (17.7)	1	1
	Medium	66 (84.6)	12 (15.4)	1.02 (0.88-1.19)	1.01 (0.87-1.17)
	Large	27 (79.4)	7 (20.6)	0.96 (0.78-1.18)	0.92 (0.76-1.12)
3.	Age (in years)			1.005 (0.99-1.01)	1.005 (0.99-1.01)
4.	Pre-operative PTA			1.003 (0.99-1.01)	1.002 (0.99-1.01)

At approximately 10 days (first postoperative assessment), 146 (83.9%) grafts were found to be in situ. At the three-week assessment, two additional graft failures were observed, leaving 144 (82.8%) grafts with successful uptake. At three months (final postoperative assessment), all 144 grafts remained intact, with no further failures. The final successful graft uptake rate was therefore 82.8%.

Hearing outcomes: Operated-ear PTA improved significantly following surgery. Mean PTA decreased from 43.76 ± 8.74 dB preoperatively to 34.95 ± 7.86 dB postoperatively, corresponding to a mean improvement of 8.81 dB (95% CI: 7.93-9.69; t (173) = 19.799, p < 0.001) (paired t-test was used).

Preoperative PTA was significantly higher in the PRP cohort than in the non-PRP cohort (45.29 ± 8.49 vs 42.23 ± 8.78 dB; mean difference 3.06 dB, 95% CI: 0.47-5.64; p = 0.021). Postoperative PTA did not differ significantly between the PRP and non-PRP cohorts (35.82 ± 7.71 vs 34.08 ± 7.95 dB; mean difference 1.74 dB, 95% CI: −0.61 to 4.08; p = 0.146). Similarly, mean PTA gain was not significantly different between the PRP and non-PRP cohorts (9.47 ± 5.51 vs 8.15 ± 6.17 dB; mean difference: 1.32 dB, 95% CI: −0.43 to 3.07; p = 0.138). In contrast, PTA gain was substantially greater among patients with successful graft uptake than among those with graft failure (10.42 ± 4.89 vs 1.07 ± 3.56 dB; mean difference 9.36 dB, 95% CI: 7.82-10.90; p < 0.001) (Table 4).

**Table 4:** Comparison of operated-ear hearing outcomes according to treatment cohort and graft uptake status (N = 174) *p-value < 0.05 was considered statistically significant. An independent-samples t-test was used. Preoperative PTA: t(172) = 2.34; postoperative PTA: t(172) = 1.47; PTA gain by treatment cohort: t(172) = 1.49; PTA gain by graft uptake status: t(172) = 9.93

Panel A: Treatment cohort				
Hearing outcome	Myringoplasty without PRP (n=87)	Myringoplasty with PRP (n=87)	Mean difference (95% CI)	p-value
Preoperative PTA (dB) Mean (± SD)	42.22 ± 8.77	45.28 ± 8.48	3.05 (0.47-5.64)	0.02*
Postoperative PTA (dB) Mean (± SD)	34.08 ± 7.95	35.82 ± 7.71	1.74 (−0.61-4.08)	0.14
PTA gain (dB) Mean (± SD)	8.15 ± 6.17	9.47 ± 5.51	1.32 (−0.43 to 3.07)	0.13
Panel B: Graft uptake status				
Hearing outcome	Successful graft uptake (n=144)	Failed graft uptake (n=30)	Mean difference (95% CI)	p-value
PTA gain (dB) Mean (± SD)	10.42 ± 4.89	1.07 ± 3.56	9.36 (7.82-10.90)	<0.001*

The hearing improvement (calculated as PTA gain) according to the graft uptake status is depicted in Figure 2. The PTA gain was substantially higher among the study participants with successful graft uptake than among those with graft failure.

**Figure 2:**
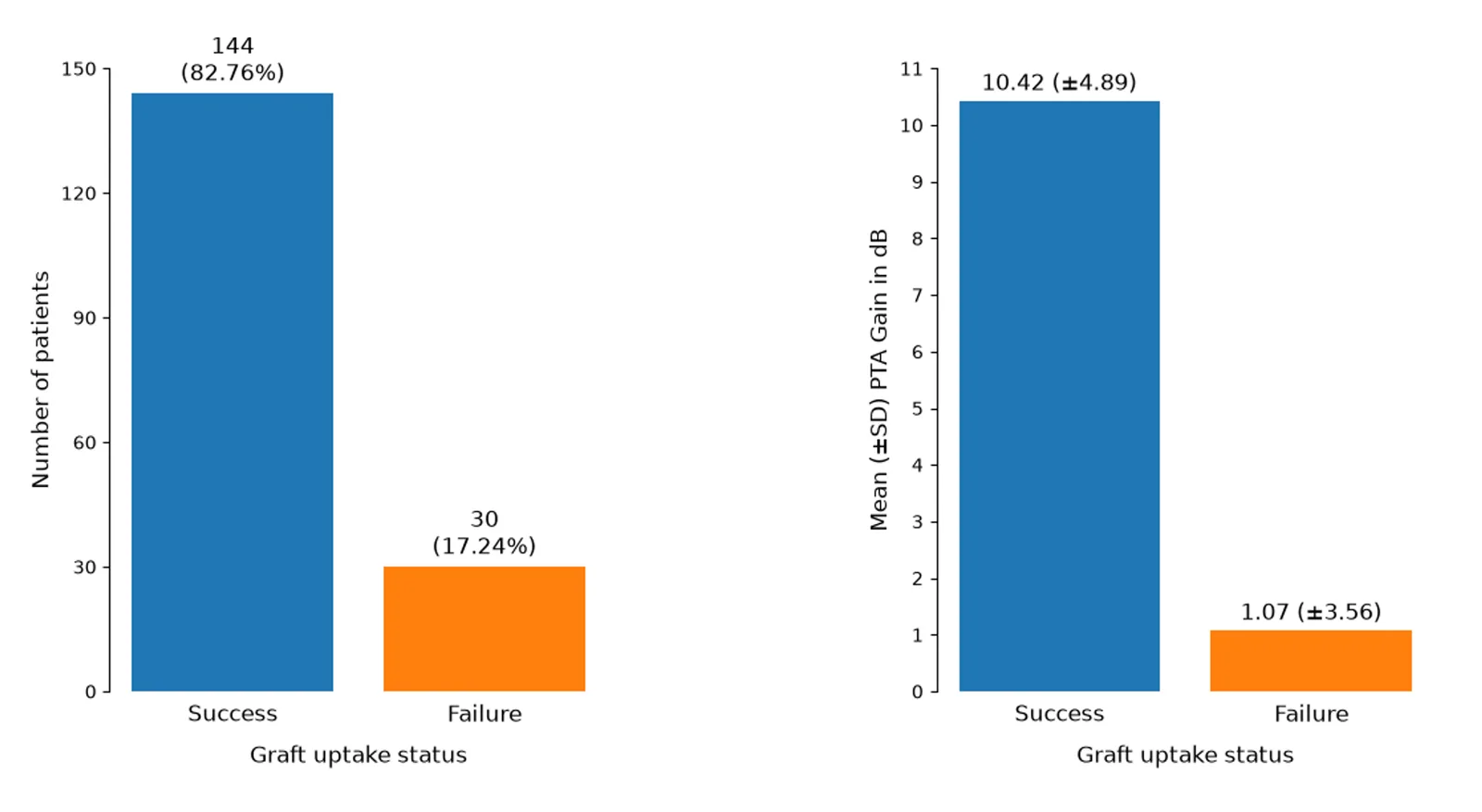
Hearing improvement according to graft uptake status (N = 174) PTA Gain: Pure-tone audiometry gain Image created by the authors using Python (version 3.12; Python Software Foundation, Beaverton, OR).

Preoperative operated-ear PTA differed between the treatment cohorts at baseline (45.29 ± 8.49 vs 42.23 ± 8.78 dB; p = 0.021); therefore, postoperative PTA was compared using ANCOVA adjusted for preoperative PTA. After adjustment for preoperative operated-ear PTA, the adjusted mean postoperative PTA was 34.77 dB (95% CI: 33.67-35.88) in the PRP cohort and 35.12 dB (95% CI: 34.02-36.23) in the non-PRP cohort. The adjusted between-cohort mean difference was −0.35 dB (95% CI: −1.92 to 1.23; p = 0.662), indicating no statistically significant difference in postoperative hearing between the treatment cohorts (Table 5).

**Table 5:** Adjusted postoperative operated-ear pure-tone audiometry (PTA) according to the treatment cohort (N = 174) *p-value < 0.05 was considered statistically significant. Analysis of covariance (ANCOVA) was used; F(1,171) = 0.19.

Treatment cohort	Adjusted postoperative PTA, mean (95% CI)	Adjusted mean difference, PRP - No PRP (95% CI)	p-value
Myringoplasty with PRP (n=87)	34.77 (33.67-35.88)	-0.35 (-1.92 to 1.23)	0.66
Myringoplasty without PRP (n=87)	35.12 (34.02-36.23)	1	

As a sensitivity analysis, multivariable binary logistic regression was performed using successful graft uptake as the dependent variable and treatment cohort, perforation size, age, and preoperative operated-ear PTA as predictors. PRP was associated with higher odds of successful graft uptake (adjusted OR: 1.997, 95% CI: 0.860-4.640), although the association was not statistically significant (p = 0.108). None of the other predictors were significantly associated with graft uptake. The Hosmer-Lemeshow test indicated no evidence of significant lack of model fit (χ² = 11.578, df = 8, p = 0.171). The direction of the association between PRP and graft uptake was consistent with the primary modified Poisson regression analysis, although the magnitude of association differed because logistic regression calculated odds ratios rather than risk ratios (Table 6).

**Table 6:** Sensitivity analysis of factors associated with successful graft uptake (N = 174) *p-value < 0.05 was considered statistically significant. The multivariable logistic regression model was used.

Sl. No.	Variables	Successful graft uptake		Adjusted OR (95% CI)	p-value
		Yes n (%)	No n (%)		
1.	Treatment cohort				
	Myringoplasty without PRP	68 (78.2)	19 (21.8)	1	-
	Myringoplasty with PRP	76 (87.4)	11 (12.6)	1.99 (0.86-4.64)	0.10
2.	Perforation size				
	Small	51 (82.3)	11 (17.7)	1	-
	Medium	66 (84.6)	12 (15.4)	1.08 (0.42-2.76)	0.87
	Large	27 (79.4)	7 (20.6)	0.62 (0.20-1.94)	0.41
3.	Age (in years)			1.03 (0.97-1.09)	0.24
4.	Pre-operative PTA			1.01 (0.96-1.06)	0.65

## Discussion

This prospective comparative cohort study evaluated the association between adjunctive autologous PRP and outcomes following myringoplasty. The principal finding was a higher graft uptake rate in the PRP cohort. However, the difference did not reach statistical significance. Hearing also improved substantially after surgery in the overall study population.

In this study, graft uptake was achieved in 87.4% of patients receiving PRP, and the corresponding rate was 78.2% without PRP. This represented an absolute difference of 9.2 percentage points, which suggests a potentially clinically meaningful improvement. However, this difference was not found to be statistically significant in this study. These findings are broadly consistent with several studies. Yadav et al. reported graft uptake of 95% with PRP and 85% with conventional underlay myringoplasty at three months, and the difference was statistically significant [19]. Vignesh et al. also reported higher graft uptake with PRP at three months, while the conventional group had a three-month graft uptake rate of 84.2% [20]. The direction of these findings is similar to the present study. The magnitude of benefit differed across studies, which could be due to several factors. The present study included small, medium, and large perforations. Differences in perforation size can influence graft healing. Larger defects generally have less favourable anatomical outcomes. Previous studies have demonstrated lower closure rates with increasing perforation size [21]. Differences in graft material, surgical approach and PRP application may further contribute to variation between studies.

A randomized trial of temporalis fascia myringoplasty reported graft uptake of 94.4% with PRP and 92.1% without PRP at six months, but the difference was not statistically significant [8]. These findings are comparable to the present study. Both studies suggest that conventional myringoplasty can achieve high closure rates. Consequently, the additional effect of PRP may become relatively small when surgical outcomes are already favourable.

A meta-analysis conducted by Huang et al. reported significantly higher TM closure rates with PRP. However, they found no significant difference in hearing improvement [6]. A more recent meta-analysis, which included 13 randomized trials and 1,179 participants, reported improved graft uptake and air-bone gap gain with PRP. However, the authors highlighted small sample sizes and inconsistent reporting among the included trials [22]. These findings support the biological plausibility of PRP while explaining why individual clinical studies may produce different results.

In this study, perforation size was not independently associated with graft uptake. This finding was different compared to previous studies. Das et al. reported markedly lower success with larger perforations and also reported success rates of 100% for small defects, 80% for medium defects [21]. A systematic review by Illés et al. similarly identified perforation size as an important predictor of the reconstruction success [23]. The absence of a significant association in this study may reflect differences in sample size, perforation classification, and patient selection. In addition, perforation size was categorized clinically as small, medium, or large in this study rather than quantified as a percentage of TM area. This may have reduced the ability to detect smaller differences between categories.

This study reported an overall improvement in PTA post-procedure, which was statistically significant. This finding supports the established functional benefit of successful TM reconstruction. Similar improvement after myringoplasty has been documented in several clinical series. A large prospective study conducted in the United Kingdom by Karela et al. reported significant hearing improvement following myringoplasty, with an overall graft success rate exceeding 90% [24]. Meta-analytic evidence also indicates that myringoplasty generally improves postoperative hearing measures [25].

However, PRP did not provide a statistically significant additional hearing benefit in this study. The mean PTA gain was 9.47 dB and 8.15 dB in the PRP cohort and non-PRP cohort, respectively. This finding agrees with the randomized study by Sharma et al., which found no significant difference in hearing improvement between PRP-assisted and conventional myringoplasty [8]. Conversely, Yadav et al. reported a mean hearing threshold gain of 18.62 dB with PRP compared with 13.15 dB in controls [19]. This discrepancy in hearing outcomes could be due to multiple factors including middle-ear mechanics, ossicular status, graft position, perforation size, and preoperative hearing, all of which influence postoperative thresholds [26]. PRP may principally influence tissue healing rather than sound-conduction mechanics, and its effect on anatomical closure may therefore be greater than its effect on hearing [27]. Differences in the definition of hearing gain may also explain the conflicting findings. Some studies have used PTA, while others have used air-bone gap or a predefined hearing-gain threshold. These measures are related but not interchangeable.

In this study, the patients with successful graft uptake had markedly greater PTA improvement than those with graft failure, which was statistically significant. The finding supports the functional importance of anatomical closure. However, this association should not be interpreted as evidence that successful graft uptake itself caused the entire hearing improvement. Both anatomical healing and postoperative hearing may be influenced by common surgical and middle-ear factors.

Neither age, perforation size, nor preoperative PTA showed a significant independent association with graft uptake in this study. These findings should be interpreted in the context of the study design. Treatment allocation was non-randomized and was influenced by participant willingness and surgeon preference, creating potential for selection bias and residual confounding. Important unmeasured factors may have influenced the decision to use PRP and the subsequent outcome. Surgeon preference itself may have been related to perceived case complexity or other clinical characteristics.

Strengths and limitations

A major strength of this study was its prospective design. Participants were enrolled before surgery and followed systematically. This allowed the temporal sequence between treatment exposure and outcomes to be maintained. The study used standardized temporalis fascia underlay myringoplasty in both cohorts, reducing heterogeneity in the principal surgical technique. Another strength was the use of objective audiological assessment (PTA), which was performed preoperatively, as well as postoperatively at three months. Hearing improvement was quantified using the PTA-gain measure. The study also used ANCOVA because baseline PTA differed between cohorts.

Several limitations should be considered. First, this was a single-centre study. The findings may therefore not be generalizable to all otological settings. Second, treatment allocation was non-randomized and based on surgeon preference. Selection bias and residual confounding therefore remain possible. Third, the PRP preparation was not characterized using quantitative platelet measurements. Variation in platelet concentration or composition may have influenced treatment response. Fourth, the operative technique and graft-related factors may vary between patients in routine clinical practice. Such variation can affect graft uptake independently of PRP. Fifth, air-bone gap outcomes were not analyzed separately. Sixth, the sample-size calculation was based on graft-uptake estimates derived from different published myringoplasty studies, which may not fully reflect the study population or surgical setting of the present cohort. Next, the perforation size was classified using quadrant-based clinical categories rather than quantitative measurement of perforation area. Finally, follow-up was limited to three months. Longer surveillance would better assess the durability of anatomical closure and hearing outcomes.

## Conclusions

Adjunctive autologous PRP was associated with a numerically higher three-month graft uptake rate, although the adjusted association was not statistically significant in this study. Both cohorts showed significant postoperative hearing improvement, with no statistically significant difference in postoperative hearing between the PRP and non-PRP cohorts. Greater hearing improvement was observed among patients achieving successful graft uptake. PRP may therefore have a potential role in promoting graft healing, but its clinical benefit remains uncertain. Larger randomized controlled studies with standardized PRP protocols and longer follow-up are required to measure the clinical benefit of autologous PRP in myringoplasty.

## Appendices

Study questionnaire

Graft Uptake Following Myringoplasty With Adjunctive Autologous Platelet-Rich Plasma: A Prospective, Non-randomized Comparative Cohort Study in Tiruvannamalai District, Tamil Nadu, South India

A. BASIC DEMOGRAPHIC DETAILS

Case No:                                                Date:

Study ID:                                               

Age (years):                                           Gender: □ Male   □ Female

Study group: □ PRP-assisted myringoplasty   □ Conventional myringoplasty

B. CLINICAL HISTORY

1. Presenting complaints:

□ Ear discharge   □ Hearing difficulty   □ Ear pain   □ Tinnitus   □ Giddiness

Other:

2. Duration of symptoms:

3. Previous ear complaints: □ Yes   □ No

4. Relevant past history:

5. Current / previous medication: □ Yes   □ No

C. EAR EXAMINATION

1. Operated ear: □ Right   □ Left

2. Perforation type/site:

3. Perforation size: □ Small   □ Medium   □ Large

4. Dry ear for ≥ 6 weeks: □ Yes   □ No

5. Clinical Eustachian tube assessment (Valsalva):

D. HEARING ASSESSMENT

1. Tuning fork findings:

Rinne's Test:                                      Weber Test:

2. Hearing type: □ Conductive   □ Mixed   □ Sensorineural

3. Pre-operative PTA (500, 1000, 2000 and 4000 Hz):

Right: __________ dB     Left: __________ dB

E. OPERATIVE DETAILS

1. Surgeon:

2. PRP used: □ Yes   □ No

If PRP used:

Blood volume:       mL     Final PRP volume:      mL

3. Anticoagulant:

4. PRP preparation/activation details:

5. Other operative remarks:

F. POSTOPERATIVE FOLLOW-UP

1. First follow-up (Approximately 10 days):

Graft status: □ In situ / satisfactory   □ Not satisfactory

Remarks:

2. Second follow-up (Approximately three weeks):

Graft status: □ Taken well   □ Not taken well

Remarks:

3. Third follow-up (Approximately three months):

Graft status: □ Intact tympanic membrane   □ Persistent / residual / recurrent perforation

Remarks:

G. FINAL STUDY OUTCOME

1. Primary outcome: graft uptake at 3 months:

□ SUCCESS: intact tympanic membrane without persistent, residual or recurrent perforation

□ FAILURE: persistent, residual or recurrent perforation

2. Secondary outcome: Post-operative hearing assessment completed: □ Yes   □ No

3. Post-operative PTA:

Right: __________ dB     Left: __________ dB

4. Any postoperative complication / adverse event: □ No   □ Yes

5. If yes, specify:

H. INVESTIGATOR'S REMARKS:

Investigator's name/signature:                                              Date:
